# International Congress of Gynæcology and Obstetrics

**Published:** 1892-03

**Authors:** 


### International Congress of Gynæcology and Obstetrics.

The first Session of this Congress, which is to meet every four years alternately in Switzerland and Belgium, will be held in Brussels from September 14th to the 19th.

The following subjects have been chosen for discussion :

1. Pelvic Suppurations, to be introduced by Dr. P. Segond of Paris.
2. Extra-uterine Pregnancy, to be introduced by Dr. A. Martin of Berlin.
3. Placenta Prævia, to be introduced by Dr. Berry Hart of Edinburgh.

The subscription for this Congress is 30 francs, which should be sent to the Secretary-General, Dr. Jacobs, 12 Rue des Petits-Carmes, Brussels, to whom any wish to read papers at the Congress should be communicated, and from whom any further information may be obtained.

